# Analysis of 665 thyroid nodules using both EU-TIRADS and ACR TI-RADS classification systems

**DOI:** 10.1186/s13044-023-00155-7

**Published:** 2023-05-08

**Authors:** Ana Paula Borges, Célia Antunes, Filipe Caseiro-Alves, Paulo Donato

**Affiliations:** 1 Radiology Department, Coimbra Hospital and Universitary Centre, Praceta Professor Mota Pinto, 3004-561 Coimbra, Portugal; 2 Faculty of Medicine of the University of Coimbra, Rua Larga 2, 3000-370 Coimbra, Portugal; 3 Academic and Clinical Centre of Coimbra, Coimbra, Portugal

**Keywords:** Thyroid Nodules, Thyroid Neoplasms, Ultrasonography, Fine-Needle Aspiration, ACR TI-RADS, EU-TIRADS

## Abstract

**Background:**

Ultrasound-based classification systems allow stratification of thyroid nodules to recommend fine-needle aspiration (FNA) based on their malignancy risk. However, these have discrepancies that may have an impact in thyroid cancer detection. We aimed to compare European Thyroid Association (EU-TIRADS) and American College of Radiology (ACR TI-RADS), in terms of FNA indication and diagnostic performance.

**Methods:**

Retrospective study of 665 thyroid nodules from 598 patients who underwent ultrasound and fine-needle aspiration at a tertiary-care institution between January 1^st^ of 2016 and July 31^st^ of 2019. Based on their sonographic features they were classified according to the EU-TIRADS and ACR TI-RADS classification and then their cytological results were obtained. Differences in FNA indications according to these two classifications were analysed. In patients who underwent surgical removal of the nodules, the final pathological diagnosis was obtained.

**Results:**

A statistically significant association was found between EU-TIRADS and ACR TI-RADS classification systems (*p* < 0.001). ACR TI-RADS allowed greatest reduction in FNA performed (32% vs 24.5%). A different risk category was obtained in 174 (26.1%) nodules, mostly higher with EU-TIRADS. The indication to FNA changed in 54 (8.1%) nodules (49 only indicated following EU-TIRADS recommendations), of which 4 had Bethesda IV and 5 had Bethesda III cytology. The FNA indication in a higher number of nodules using EU-TIRADS was due to difference in the dimensional threshold for FNA on low-risk nodules; to the fact that hypoechogenicity in a mixed nodule ascribes it moderate risk, while using ACR TI-RADS it would only be considered of low risk, and to the use of isolated sonographic features, namely marked hypoechogenicity, microcalcifications and irregular margins, to automatically categorize a nodules as high risk in EU-TIRADS, while ACR TI-RADS requires a group of potentially suspicious features to consider a nodule of high risk. The analysis of pathology proven nodules revealed equally good sensitivity of both systems in the detection of malignancy, but weak specificity, slightly greater with ACR TI-RADS (27.1% vs 18.6%).

**Conclusions:**

The EU-TIRADS and ACR TI-RADS are both suitable to assess thyroid nodules and through risk stratification avoid unnecessary FNA. FNA was less performed using ACR TI-RADS, which was slightly more efficiency in excluding malignancy.

## Background

Thyroid nodules are more and more often detected given the increasing use of imaging (about 41% of the population by ultrasound), Mostly incidentally and asymptomatic and only 10% malignant [1, 2].

Ultrasound is the most precise and cost-effective in their evaluation, whose objective is to distinguish between those benign that may be kept under surveillance from those with malignant features that require additional approaches. Fine-needle aspiration (FNA) has revealed high sensitivity and specificity in that distinction, with a rate of non-diagnostic results of only 2–16%. However, its performance must be selective in order to avoid unnecessary surgeries, which are not risk-free in nodules with indeterminate cytology (5–20%), of which only 20% are malignant. Thus, FNA indications should be based on sonographic stratification of the risk of malignancy, in conjunction with the clinical presentation and patient’s risk factors [1, 3, 4].

To balance the benefit of detecting clinically significant cancers with the risk and cost of FNA and treatment of benign nodules or indolent cancers, classification systems have arisen to group nodules in categories with equal percentage of malignancy risk, based on sonographic features. An ideal stratification system must recommend the least number of FNA possible, identifying most neoplasms [5].

The initial propose of Thyroid Imaging Reporting and Data System (TI-RADS) was developed in 2009 by Horvath et al*.*, and later others emerged, the American College of Radiology (ACR) Thyroid Imaging Reporting and Data System (ACR TI-RADS) and the European Thyroid Imaging and Reporting Data System (EU-TIRADS) being among the most used. Their intent was to simplify the report of sonographic findings [2, 3, 6, 7].

**Fig. 1:**
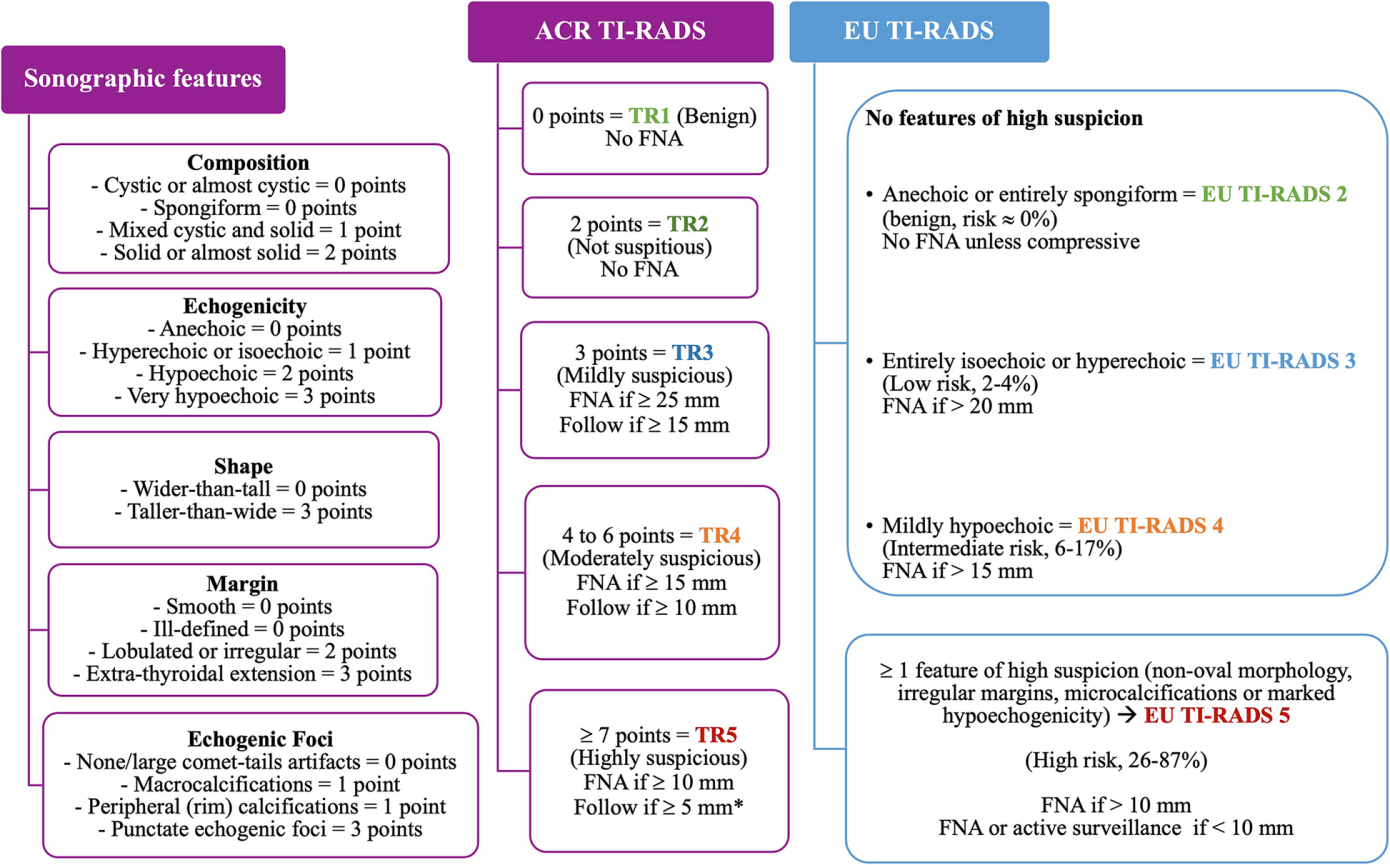
Differences between ACR TI-RADS and EU-TIRADS classification systems. In ACR TI-RADS, the risk category is based on a sum of points assigned to 5 sonographic features. In EU-TIRADS a specific feature instantly classifies the nodule into one category. Adapted from references 3 and 6. Adapted from references 3 and 6

These systems share some characteristics, but they also have some differences (Fig. 1). In ACR TI-RADS the nodules receive a sum of points assigned to 5 sonographic features (composition, echogenicity, morphology, margins, and echogenic foci). Based on their final score, one of five risk category is assigned, from TR1 (benign) to TR5 (highly suspicious of malignancy). In EU-TIRADS a specific feature instantly classifies the nodule into one category, eliminating the need of point summation and consequently simplifying the classification process and turning it less time-consuming. It only takes the presence of non-oval morphology, irregular margins, microcalcifications or marked hypoechogenicity to consider a nodule of high risk of malignancy (category 5). Inherent to its greater simplicity is the non-consideration of some potentially relevant features, namely the presence of macrocalcifications [3, 6].

The goal of this study was to compare the performance of EU-TIRADS and ACR TI-RADS in terms of avoidance of unnecessary FNA and diagnostic performance in thyroid nodules submitted to ultrasound and FNA.

## Methods

A retrospective analysis of thyroid nodules submitted to ultrasound and FNA at a tertiary-care institution between January 1^st^ of 2016 and July 31^st^ of 2019 was performed. Demographic data (sex and age) of patients were registered and sonographic images of the nodules available in the Hospital’s archive were analysed, without previous knowledge of the histological results. Ultrasound and FNA were performed by four different radiologists with 3 to 20 years of experience in thyroid imaging.

To each nodule the sonographic features evaluated included its size (largest axis in millimetres), location (right lobe, left lobe, isthmus), composition (cystic/ almost completely cystic, spongiform, mixed or solid/almost completely solid), echogenicity (anechoic, very hypoechoic, hypoechoic, isoechoic or hyperechoic relatively to the adjacent thyroid parenchyma), morphology (taller-than-wide or not), contour (smooth, ill-defined, lobulated/irregular, extra-thyroidal extension), presence of echogenic foci (none, with comet-tail artifact, macrocalcifications, peripheral calcifications or punctate echogenic foci) and the presence or absence of hypoechoic halo. In nodules located both in a lobe and isthmus its predominant location was considered. In nodules with mixed composition, the echogenicity of its solid component was considered. Based on these features, each nodule was classified according to ACR TI-RADS and EU-TIRADS, by means of retrospective analysis of the sonographic images. Subsequently, cytological results were obtained, including its classification by Bethesda System [8]. In surgically excised nodules (within a maxim period of three months after sonographic evaluation), the histopathological result was obtained.

Nodules < 10 mm (without indication to FNA in neither classification system) and those with non-diagnostic ytological results were excluded. Lastly, differences between FNA recommendations of both systems were analysed.

Statistical analysis was performed with the 23^rd^ version of the *Statistical Package for Social Science* (SPSS). Chi-square (χ2) and Fisher exact tests were used to correlate categorical variables, as indicated. It was considered statistically significant a p-value < 0.05.

This retrospective study was approved by our institution’s Ethics Committee and the requirement for informed consent from patients was waived.

## Results

### Study sample

During study’s period, a total of 701 thyroid nodules of 646 patients were submitted to ultrasound and FNA. Eleven nodules under 10 mm were excluded, as were 25 with non-diagnostic cytological results. The final sample included 665 nodules in 598 patients, 556 females (83.6%) and 109 males (16.4%), with an average (± Standard Deviation) age of 59.1 ± 15.6 years old.

**Fig. 2:**
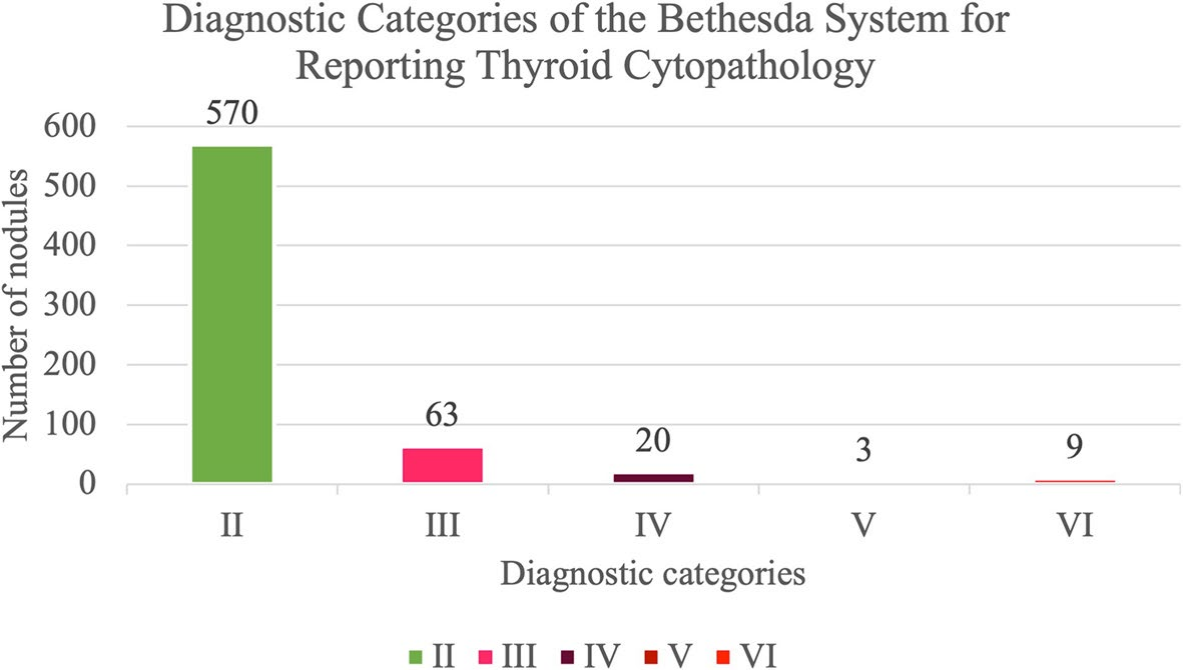
Diagnostic Categories of the Bethesda System for Reporting Thyroid Cytopathology

Cytological result according to was atypia of undetermined significance/follicular lesion of undetermined significance (AUS/FLUS) in 63 (9.5%), follicular neoplasm or suspicious for a follicular neoplasm in 20 (3%), suspicious for malignancy in 3 (0.5%), malignant in 9 (1.4%), and benign in the remainder 570 (85.7%), mainly colloid or hyperplasic adenomatous nodules. The proportion of nodules within each diagnostic category of the Bethesda System for Reporting Thyroid Cytopathology is represented in Fig. 2. Descriptive analysis of sonographic features of thyroid nodules is detailed in Table 1.

**Table 1:** Descriptive analysis of sonographic features of thyroid nodules

Sonographic features	Thyroid nodules (*n* = 665)
Size in mm (average ± SD)	24.5 ± 10.9
Location	
Right lobe	316 (47.5)
Left lobe	319 (48)
Isthmus	30 (4.5)
Composition	
Cystic/ almost completely cystic	17 (2.6)
Spongiform	22 (3.3)
Mixed	103 (15.5)
Solid/almost completely solid	523 (78.6)
Echogenicity	
Anechoic	17 (2.6)
Very hypoechoic	86 (12.9)
Hypoechoic	382 (57.4)
Isoechoic	154 (23.2)
Hyperechoic	26 (3.9)
Morphology	
Taller-than-wide	43 (6.5)
Other	622 (93.5)
Contour	
Smooth	576 (86.6)
Ill-defined	30 (4.5)
Lobulated/irregular	43 (6.5)
Extra-thyroidal extension	16 (2.4)
Echogenic foci	
None/with comet-tail artifact	539 (79.5)
Macrocalcifications	86 (12.9)
Peripheral calcifications	9 (1.4)
Punctate echogenic foci	41 (6.2)
Bethesda classification	
II	570 (85.7)
III	63 (9.5)
IV	30 (3)
V	3 (0.5)
VI	9 (1.4)

Categorical variables are expressed in number (%)

**Fig. 3:**
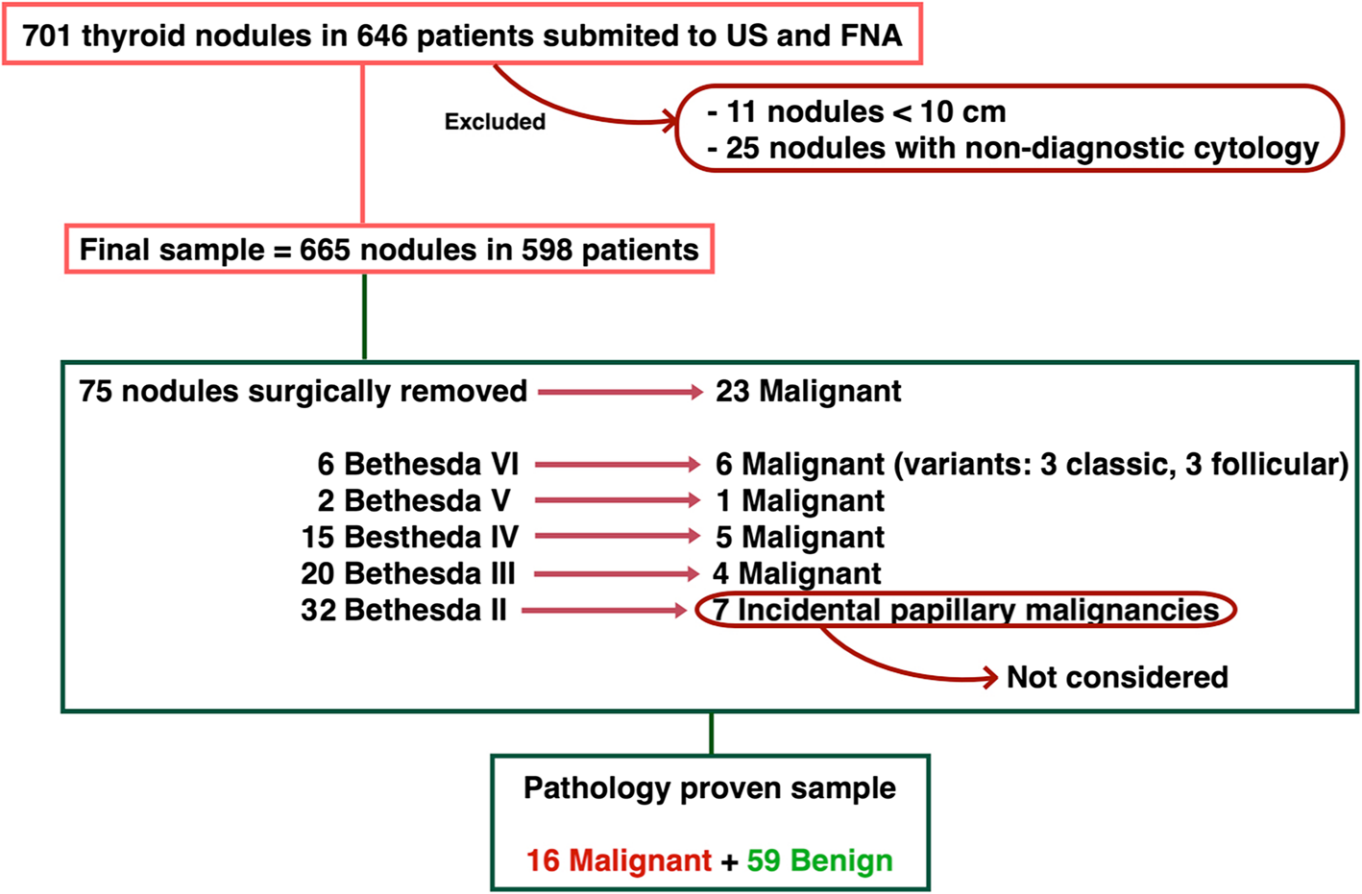
Final study sample selection after applying exclusion criteria (size < 10 mm, non-diagnostic cytology). AUS/FLUS = atypia of undetermined significance/follicular lesion of undetermined significance

Of the 665 nodules studied, 75 were surgically removed, including 6 Bethesda VI, 2 Bethesda V, 15 Bethesda IV, 20 Bethesda III, and 32 Bethesda II nodules. All specimens were evaluated by the same pathology team. Malignancy was reported in 23 of them, which included 6 Bethesda VI (3 classic and 3 follicular variant), 1 Bethesda V (classic variant papillary carcinoma), 5 Bethesda IV, 4 Bethesda III (follicular carcinoma) and 7 Bethesda II (incidental papillary tumours). Therefore, in the analysis, the malignant group includes the 16 surgically confirmed (excluding the incidental tumours) (Fig. 3).

Presumed reasons for the surgical resection of nodules without malignant histology include negative compressive or aesthetic effects (their average size was 40 mm), or fear of malignancy.

Notably, among the nodules with non-diagnostic citology histology (*n* = 25) not included in the analysis, an incidental papillary carcinoma was found in the specimen.

## Comparison between ACR and EU TI-RADS

**Fig. 4:**
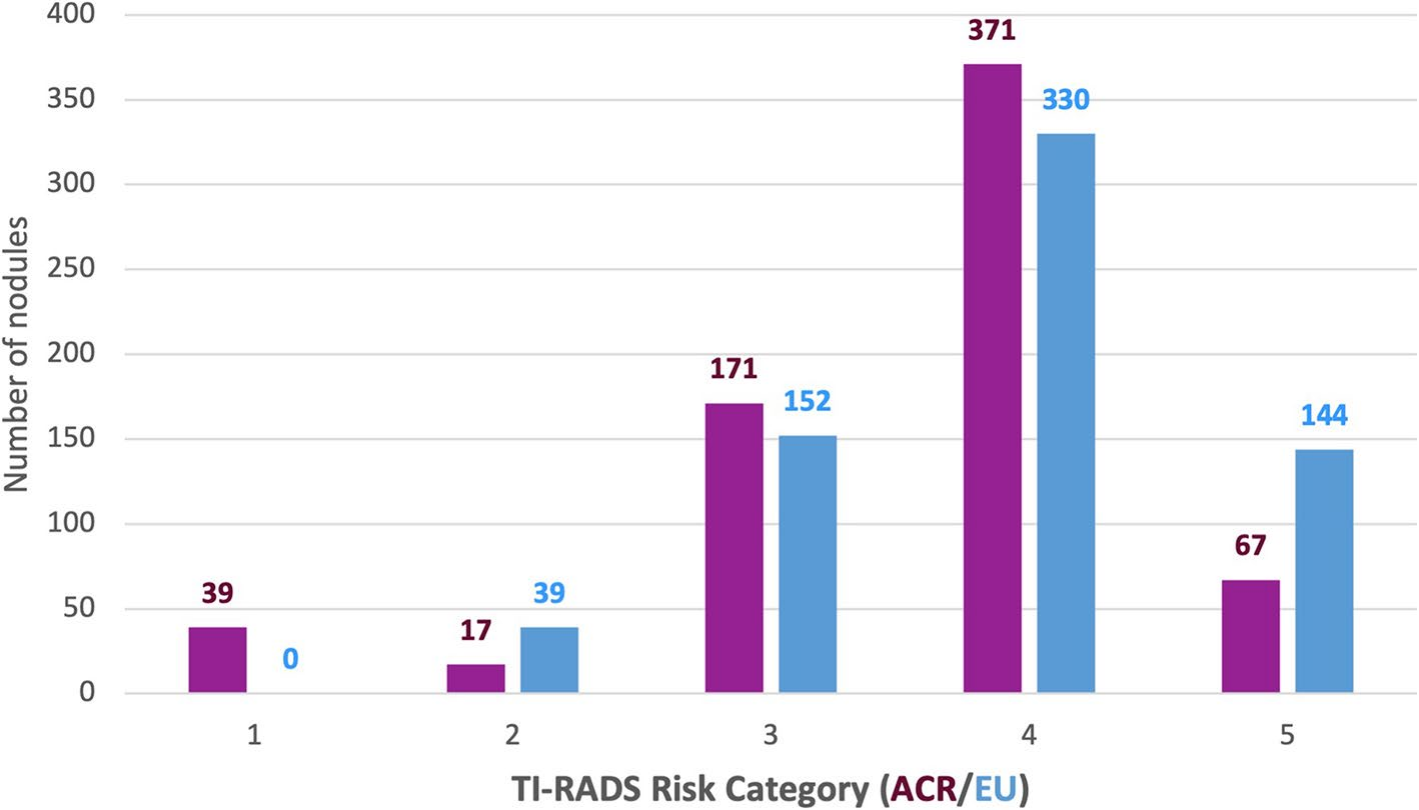
Risk Categories of the thyroid nodules assigned according to ACR TI-RADS (purple) and EU-TIRADS (blue)

A statistically significant association was found between equivalent risk categories of ACR TI-RADS and EU-TIRADS (p < 0.001). Categories obtained with both systems are represented in Fig. 4. Using EU-TIRADS the number of nodules classified as TI-RADS 5 was higher (*n* = 144) in comparison with ACR TI-RADS (*n* = 67).

Regarding recommendations to perform FNA according to the nodules’ size, there would have been a reduction of 32% (*n* = 213) and 24.5% (*n* = 163) in FNA performed according to ACR TI-RADS and EU-TIRADS, respectively.

Both ACR and EU-TIRADS classification systems presented a statistically significant association with Bethesda classification levels < III/$\ge$ III (*p* = 0.005 and *p* < 0.001, respectively, Table 2).

**Table 2:** Correlation between ACR and EU-TIRADS classification systems and Bethesda classification levels < III/$\ge$ III

	**Bethesda < III**	**Bethesda** $\ge$ **III**	***p*-value**
ACR TIRADS			0.005
1	38	1	
2	16	1	
3	151	20	
4	316	55	
5	49	95	
EU-TIRADS			< 0.001
2	38	1	
3	128	24	
4	294	36	
5	110	34	

**Fig. 5:**
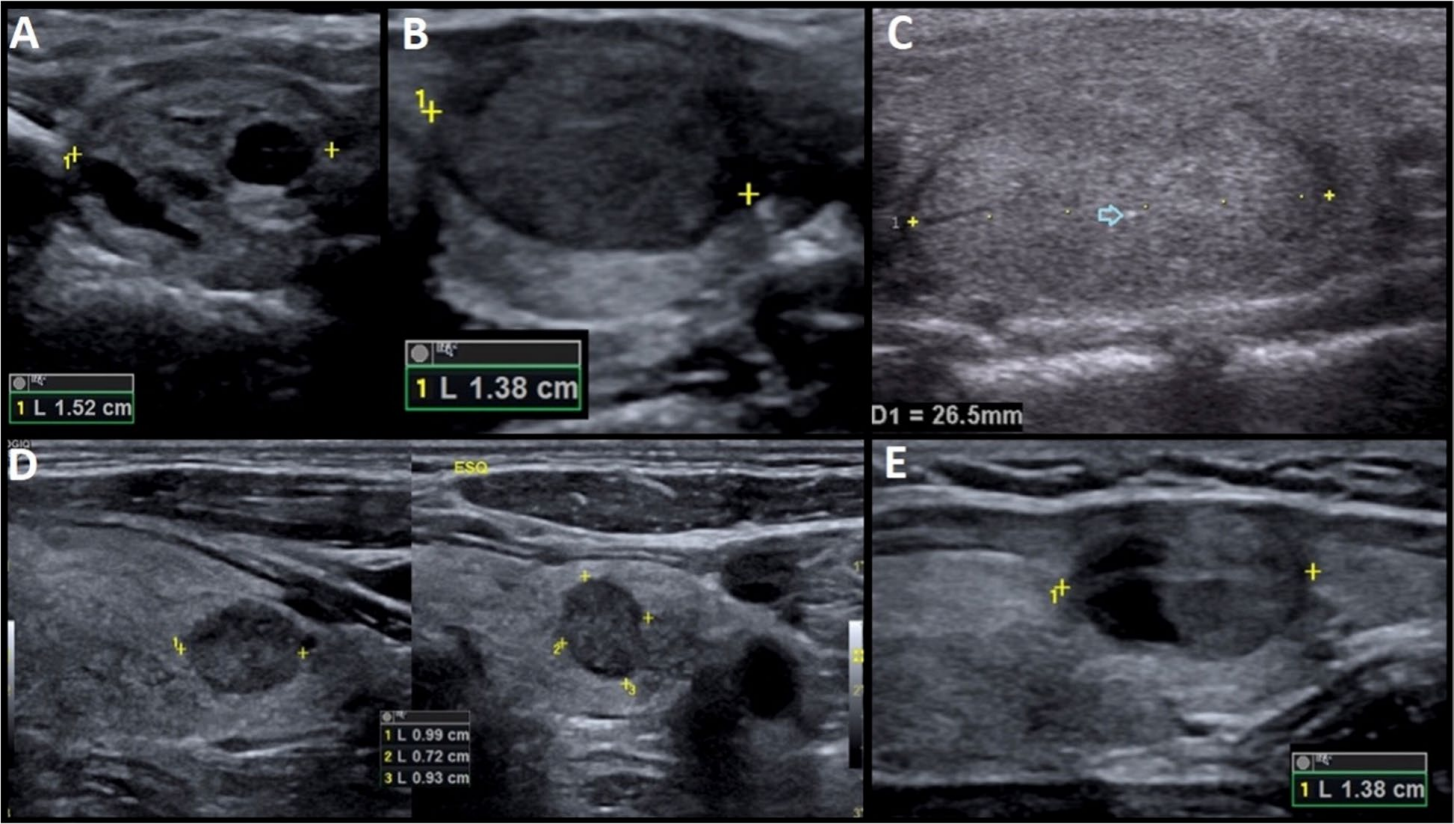
Representative images of nodules with different risk categories according to ACR TI-RADS and EU-TIRADS. **A** – nodule with mixed composition with isoechoic solid component (category ACR 2/EU 3); **B** – solid nodule markedly hypoechoic (category ACR 4/ EU 5); **C** – solid isoechoic nodule with punctate echogenic foci (arrow, category ACR 4/EU 5); **D** – solid hypoechoic nodule with taller-than-wide morphology (category ACR 4/ EU 5); **E** – solid hypoechoic nodule with posterior bulging of thyroid’s contour, indicative of extrathyroidal extension (category ACR 5/EU 4)

Excluding risk category EU-TIRADS 1 and combining categories ACR TI-RADS 1 and 2 in a category of benign or non-suspicious nodules, equivalent to the category EU-TIRADS 2, a different risk category was obtained in 174 nodules (26.1%), mainly with an upgrade of the EU-TIRADS category (Table 3, Fig. 5). Regarding the size of the nodules, that discrepancy would change the indication to perform FNA in 54 (8.1%). In 49 of those, FNA would only be indicated following EU-TIRADS recommendations, whereas in 5 it would only be indicated following ACR TI-RADS. In the remaining 491 nodules (73.8%), the level of risk was identical independently of the classification system applied.

**Table 3:** Sonographic features of nodules that obtained different risk categories according to ACR TI-RADS and EU-TIRADS

Number of nodules	Sonographic features	ACR/EU TI-RADS classification	Recommendation to FNA	Number of nodules punctured	Recommendation discrepancy
17	Mixed Isoechoic	2/3	ACR: none EU: > 20 mm	ACR: 0 EU: 12	12 only EU - 1 Bethesda IV - 11 Bethesda II (7 removed = benign, 2 incidental papillary tumours)
55	Mixed Hypoechoic (Fig. 5A)	3/4	ACR: ≥ 25 mm EU: > 15 mm	ACR: 31 EU: 49	18 only EU - 1 Bethesda IV - 17 Bethesda II
59	Solid/Mixed Very hypoechoic (Fig. 5B)	4/5	ACR: ≥ 15 mm EU: > 10 mm	ACR: 44 EU: 59	15 only EU - 3 Bethesda III - 12 Bethesda II
8	Solid Isoechoic Or Mixed Hypoechoic + Punctate echogenic foci (Fig. 5C)	4/5	ACR: ≥ 15 mm EU: > 10 mm	ACR: 6 EU: 8	2 only EU - 1 Bethesda IV (removed, incidental papillary tumour) - 1 Bethesda II
4	Solid Iso-/Hyperechoic Irregular margins	4/5	ACR: ≥ 15 mm EU: > 10 mm	ACR: 2 EU: 4	2 only EU - 2 Bethesda II
15	Solid Isoechoic Macrocalcifications	4/3	ACR: ≥ 15 mm EU: > 20 mm	ACR: 14 EU: 9	5 only ACR - 1 Bethesda IV (removed, benign) - 2 Bethesda III (removed, benign) - 2 Bethesda II
7	Solid Iso-/Hyperechoic Or Mixed isoechoic + Taller-than-wide	4/5	ACR: ≥ 15 mm EU: > 20 mm	ACR: 6 EU: 6	-
4	Solid Isoechoic Extrathyroidal extension	4/3	ACR: ≥ 15 mm EU: > 20 mm	ACR: 3 EU: 3	-
5	Solid Hypoechoic Extrathyroidal extension (Fig. 5D)	5/4	ACR: ≥ 10 mm EU: > 15 mm	ACR: 5 EU: 5	-
39	Cystic/Spongiform	1/2	ACR: none EU: none	-	-

Only 4 of the cases with a discrepancy in the recommendation to perform FNA (3 only indicated following EU TI-RADS and 1 following ACR TIRADS) had Bethesda IV cytology, of which only 2 were surgically removed, with benign results (although an incidental papillary tumour was found). Five nodules with similar discrepancy (3 only indicated following EU TI-RADS and 2 following ACR TIRADS) had Bethesda III cytology, of which only one was removed, with benign results. In the remaining nodules, when the indication to perform FNA was different, the cytological results were Bethesda II, of which 7 were surgically removed. Interestingly, in 2 of those (with FNA only recommended following EU-TIRADS), incidental papillary microcarcinomas in surgical specimens.

Considering only the 75 nodules that were surgically removed (16 malignant and 59 benign), only 2 of the 18 nodules that wouldn’t have indication to perform FNA according to ACR TI-RADS proved to be malignant (11.1% false negatives) and 14 of the 57 nodules with indication (24.6% true positives). Following EU-TIRADS recommendations, 2 of the 13 nodules without indication to FNA were malignant (15.4% false negatives), as well as 14 of the 62 with indication (22.6% true positives). One papillary carcinoma (classic variant) wouldn’t be detected following both system’s recommendations (isoechoic solid nodule, with no other suspicious features).

Sensitivity, specificity, positive predictive value (PPV), negative predictive value (NPV) and accuracy of both classification systems in the detection of malignant thyroid nodules are represented in Table 4. Both exhibit a good sensitivity and NPV but weak specificity and PPV. ACR TI-RADS turned out to be somewhat more specific (27.1% vs 18.6%) and accurate (40% vs 33.3%). Thus, ACR TI-RADS appears more efficient in the exclusion of malignancy, although the difference between the performance of both is small.

**Table 4:** Sensitivity, specificity, PPV, NPV and accuracy of ACR TI-RADS and EU-TIRADS, according to FNA recommendation

	**ACR TI-RADS**		**EU-TIRADS**	
	**Nodules with recommendation to FNA**	**Nodules without recommendation to FNA**	**Nodules with recommendation to FNA**	**Nodules without recommendation to FNA**
**Malignant (*n* = 16)**	14 (TP)	2 (FN)	14 (TP)	2 (FN)
**Benign (*n* = 59)**	43 (FP)	16 (TN)	48 (FP)	11 (TN)
**Total (*n* = 75)**	57	18	62	13
**Sensitivity**	87.5%		87.5%	
**Specificity**	27.1%		18.6%	
**PPV**	24.6%		22.6%	
**NPV**	88.9%		84.6%	
**Accuracy**	40%		33.3%	

*FN* False negatives, *FNA* Fine-needle aspiration, *FP* False positives, *NPV* Negative predictive value, *PPV* Positive predictive value, *TN* True negatives, *TP* True positives

## Discussion

Ultrasound is indicated in the initial evaluation and follow-up of thyroid nodules. Classification and malignancy risk stratification systems based on sonographic features of those nodules allow improvement in interobserver reproducibility in their description, simplification in communicating the results, and aiding in the decision whether to perform FNA or not, minimizing unnecessary punctions [3]. ACR TI-RADS incorporates all the sonographic features of the nodules, scored based on their malignant potential, making this system objective and detailed. EU-TIRADS is simpler, including only 4 sonographic features indicative of a high risk of malignancy.

A common limitation to both classification systems is the potential difficulty in interpreting some of the nodules’ features, as the identification of a spongiform composition and distinguishing between punctate echogenic foci that represent microcalcifications from comet-tail artifacts that reflect colloid crystals. However, the agreement in the final TI-RADS category is usually more consistent between readers than the agreement regarding individual features. To reduce the interobserver variability, radiologists’ meetings to discuss discrepant cases and obtaining second opinions in cases of uncertainty could be useful [3, 6, 9].

Regarding nodules’ size, although there are studies in which larger nodules were associated with a higher probability of malignancy, others revealed an inverse association with the size [10–12]. Thus, nodule size is not considered useful in distinguishing between benign and malignant nodules, and in both ACR TI-RADS and EU-TIRADS, it is a criterion to recommend FNA but not to stratify the risk of malignancy [3, 6, 13]. In fact, FNA of TR5 nodules with 5 to 9 mm in size may be appropriate under some circumstances, and the decision to perform FNA must be based not only on the nodule’s size but also on the clinical risk factors (for example, it doesn't make sense in inoperable patients, or those with a low life expectancy because of other comorbidities) and patient’s desire [3, 6].

Considering a nodule with mixed echogenicity, ACR suggests that it should be described as “predominantly” hiper-, iso-, or hypoechoic. In EU-TIRADS, it is the echogenicity of the solid component that distinguishes between low risk and intermediate-risk categories (it only takes any hypoechoic portion to be considered intermediate-risk, as it is only required a very hypoechoic portion to be considered high-risk). Of the nodules with a different ACR TI-RADS and EU-TIRADS classification in our study, in most cases (75.3%, *n* = 131) such discrepancy was due to the nodule’s echogenicity, with a higher risk category using EU-TIRADS. Among those, FNA was only recommended according to EU-TIRADS in 45, two of which proved had Bethesda IV cytology (not surgically removed for pathology confirmation). The remaining were benign, with 2 incidental papillary tumours being found in surgical specimens. Despite the consideration of the echogenicity alone increased the risk category in EU-TIRADS (thus reducing the dimensional cut-off to recommend FNA), the authors of this system make the reservation that this feature must be combined with the presence of other features that reduce or increase the risk of malignancy [3].

Both ACR and ETA consider microcalcifications a feature of high risk of malignancy. ACR TI-RADS assigns 3 points to the nodule and EU-TIRADS categorize as risk category 5, regardless of its other features. For this reason, the presence of such foci in 8 nodules of our study, considered moderately suspicious according to ACR TI-RADS, turned them of high-risk according to EU-TIRADS. Two of them would only have an indication to perform FNA according to EU-TIRADS, one of which had Bethesda IV cytology, whose removal revealed only an incidental papillary tumour.

The point assigned to the presence of macrocalcifications in ACR TI-RADS, contributed to the fact that 15 solid isoechoic nodules were considered moderately suspicious, whereas according to EU-TIRADS they were low-risk. Five of them would only have an indication to perform FNA according to ACR TI-RADS, one of them with Bethesda IV The distinction between irregular and lobular margins is a matter of discussion, representing the sonographic feature with greater interobserver variability [14]. Despite that, the presence of an irregular margin is a highly precise marker of malignancy. ACR TI-RADS assigns 2 points to the nodules, whereas EU-TIRADS puts it automatically in a high-risk category. For this reason, 4 of the analyzed nodules obtained an ACR TI-RADS 4 e EU-TIRADS 5 classification (all benign).

Both ACR and ETA consider the presence of extrathyroidal extension (bulging, protrusion or disruption of the capsular margin) a highly reliable sign of malignancy and unfavorable prognosis. Although it only assigns 4 points in the ACR TI-RADS, it is not included in the objective criteria of risk stratification in EU-TIRADS [3, 6]. For this reason, 9 nodules with capsular bulging in our study had a higher risk category with ACR TI-RADS. None of them was malignant.

Regarding nodular morphology, ACR considers that the taller-than-wide appearance is a poorly sensitive indicator of malignancy, but highly specific. It seems to be due to the compressibility of the nodule, reflecting a centrifugal growth, as opposed to the growth of benign nodules, parallel to the normal thyroid tissue plane [12, 15]. In ACR TI-RADS 3 points are assigned to taller-than-wide nodules, which are instantaneously considered high risk in EU-TIRADS. For this motive, 7 nodules in our study had a higher risk category with EU-TIRADS, without discrepancy in FNA indication.

In the present study, both systems allowed a significant reduction in the number of FNA performed (specially ACR TI-RADS), at the cost of non-diagnosing very few malignant nodules. The authors of a prospective study with 502 nodules that showed similar results suggest that the greater reduction of FNA with ACR TI-RADS reflects the greater dimensional cut-off to recommend it in the low-risk nodules (25 mm instead of 20 mm). Such value was chosen given the fact that follicular cancers < 2 cm rarely present with distant metastasis [6, 16]. In our study, that was the case of 17 nodules low-risk in both classifications, measuring between 20 and 25 mm, thus only having indication to perform FNA according to EU-TIRADS.

As previously mentioned, the main cause of discrepant categories, responsible for a greater category according to EU-TIRADS and consequently indication to perform FNA in a greater number of nodules using this system, was the fact that the hypoechogenicity of a mixed nodule confers it moderate risk, whereas according to ACR TI-RADS it would have been considered only low-risk. Another explanation for the discrepancy is the application of isolated sonographic features, namely very low echogenicity, presence of microcalcifications, and irregular margins to directly assign a high-risk category to a nodule in EU-TIRADS (resulting in a greater number of nodules with this category), whereas in ACR TI-RADS it is necessary a group of potentially suspicious features to consider a nodule of high-risk. It is important to state that, among nodules with indication to perform FNA only according to EU-TIRADS, only 3 revealed Bethesda IV cytology, of which only one was surgically removed, whose specimen revealed an incidental papillary tumour (as was the case of the one nodule with indication to perform FNA only according to ACR TIRADS, whose specimen revealed benignity). Notably, 2 incidental papillary microcarcinomas were found in surgical specimens of after benign cytology of nodules with indication to perform FNA only according to EU-TIRADS.

A systematic review and meta-analysis of 29 studies comparing the diagnostic performance in the detection of thyroid cancer of four US-based risk stratification systems in a total of 33 748 thyroid nodules reported a higher pooled sensitivity (with a larger difference considering only category 5 nodules) and slightly lower specificity for EU-TIRADS, compared with ACR TIRADS, although the overall diagnostic performance of all systems was comparable [17]. Our results show equally good sensitivity and NPV between ACR TIRADS and EU-TIRADS, although ACR TI-RADS was slightly more specific (27.1% vs 18.6%) and accurate (40% vs 33.3%). Still, the differences found between the performance of both were very small.

## Limitations

The main limitations of this study include its retrospective nature, limiting the categorization of nodules to the sonographic images available, as well as the fact that the sonographic evaluation was performed by different operators and ultrasound machines, which could have influenced the image-based nodule characterization and classification and consequently, the results. Besides, the initial sample was comprised by a group of patients referred by doctors of different specialties to perform FNA and may therefore not constitute a representative sample of the general population. The number of pathology proven nodules was small, of which few were malignant, although with a relatively similar proportion of follicular and papillary carcinomas. These limitations are overcome with larger size controlled prospective studies.

## Conclusions

Classification and malignancy risk stratification systems of thyroid nodules based on sonographic features allow the limitation of FNA performance to the really necessary cases, in a uniformized and simplified manner. Data analysed in this study revealed a statistically significant association between ACR TI-RADS and EU-TIRADS and both can be considered appropriate to stratify malignancy risk of thyroid nodules. ACR TI-RADS allowed a greater reduction of FNA performed, without significant loss in the detection of malignant nodules.

## Data Availability

Data sharing is not applicable to this article as no datasets were generated or analysed during the current study.

## Abbreviations

ACR TI-RADS: American College of Radiology Thyroid Imaging Reporting and Data System
AUS/FLUS: Atypia of undetermined significance/follicular lesion of undetermined significance
ETA: European Thyroid Association
EU-TIRADS: European Thyroid Association Thyroid Imaging Reporting and Data System
FNA: Fine-needle aspiration
NPV: Negative predictive value
PPV: Positive predictive value
SPSS: Statistical Package for Social Science

## References

[1] Dean DS, Gharib H (2008). Epidemiology of thyroid nodules. Best Pract Res Clin Endocrinol Metab.

[2] Rahal AJ, Falsarella PM, Rocha RD, Lima JPBC, Iani MJ, Vieira FAC (2016). Correlation of Thyroid Imaging Reporting and Data System [TI-RADS] and fine needle aspiration: experience in 1,000 nodules. Einstein (Sao Paulo, Brazil).

[3] Russ G, Bonnema SJ, Erdogan MF, Durante C, Ngu R, Leenhardt L (2017). European thyroid association guidelines for ultrasound malignancy risk stratification of thyroid nodules in adults: the EU-TIRADS. Eur Thyroid J.

[4] Paschke R, Cantara S, Crescenzi A, Jarzab B, Musholt TJ, Sobrinho SM (2017). European thyroid association guidelines regarding thyroid nodule molecular fine-needle aspiration cytology diagnostics. Eur Thyroid J.

[5] Fish SA (2019). ACR TIRADS is best to decrease the number of thyroid biopsies and maintain accuracy. Clinical Thyroidol.

[6] Tessler FN, Middleton WD, Grant EG, Hoang JK, Berland LL, Teefey SA (2017). ACR thyroid imaging, reporting and data system (TI-RADS): white paper of the ACR TI-RADS committee. J Am Coll Radiol.

[7] Horvath E, Majlis S, Rossi R, Franco C, Niedmann JP, Castro A (2009). An ultrasonogram reporting system for thyroid nodules stratifying cancer risk for clinical management. J Clin Endocrinol Metab.

[8] Cibas ES, Ali SZ (2009). The bethesda system for reporting thyroid cytopathology. Am J Clin Pathol.

[9] Tappouni RR, Itri JN, McQueen TS, Lalwani N, Ou JJ (2019). ACR TI-RADS: pitfalls, solutions, and future directions. Radiographics.

[10] Arpana, Panta OB, Gurung G, Pradhan S (2018). Ultrasound Findings in Thyroid Nodules: A Radio-Cytopathologic Correlation. J Medi Ultrasound..

[11] Cappelli C, Castellano M, Pirola I, Cumetti D, Agosti B, Gandossi E (2007). The predictive value of ultrasound findings in the management of thyroid nodules. QJM..

[12] Moon W-J, Jung SL, Lee JH, Na DG, Baek J-H, Lee YH (2008). Benign and malignant thyroid nodules: us differentiation—multicenter retrospective study. Radiology.

[13] Moon W-J, Baek JH, Jung SL, Kim DW, Kim EK, Kim JY (2011). Ultrasonography and the ultrasound-based management of thyroid nodules: consensus statement and recommendations. Korean J Radiol.

[14] Anil G, Hegde A, Chong FHV (2011). Thyroid nodules: risk stratification for malignancy with ultrasound and guided biopsy. Cancer Imag.

[15] Kim EK, Park CS, Chung WY, Oh KK, Kim DI, Lee JT, et al. New sonographic criteria for recommending fine-needle aspiration biopsy of nonpalpable solid nodules of the thyroid. AJR Am J Roentgenol. 2002;178(3):687–91. 10.2214/ajr.178.3.1780687 11856699

[16] Grani G, Lamartina L, Ascoli V, Bosco D, Biffoni M, Giacomelli L, et al. Reducing the Number of Unnecessary Thyroid Biopsies While Improving Diagnostic Accuracy: Toward the "Right" TIRADS. J Clin Endocrinol Metab. 2019;104(1):95–102. 10.1210/jc.2018-01674 30299457

[17] Kim PH, Suh CH, Baek JH, Chung SR, Choi YJ, Lee JH. Diagnostic Performance of Four Ultrasound Risk Stratification Systems: A Systematic Review and Meta-Analysis. Thyroid. 2020;30(8):1159-68. 10.1089/thy.2019.0812. 10.1089/thy.2019.0812 32303153

